# The FOCUS, AFFINITY and EFFECTS trials studying the effect(s) of fluoxetine in patients with a recent stroke: statistical and health economic analysis plan for the trials and for the individual patient data meta-analysis

**DOI:** 10.1186/s13063-017-2385-6

**Published:** 2017-12-28

**Authors:** Catriona Graham, Steff Lewis, John Forbes, Gillian Mead, Maree L. Hackett, Graeme J. Hankey, John Gommans, Huy Thang Nguyen, Erik Lundström, Eva Isaksson, Per Näsman, Ann-Sofie Rudberg, Martin Dennis

**Affiliations:** 1 Welcome Trust Clinical Research Facility, Edinburgh, UK; 2 Edinburgh Clinical Trials Unit, University of Edinburgh, Edinburgh, UK; 3 Health Research Institute, University of Limerick, Limerick, Ireland; 4 Centre for Clinical Brain Sciences, University of Edinburgh, Chancellors Building FU303h, 49 Little France Crescent, Edinburgh, EH16 4SB UK; 5 The George Institute for Global Health, University of Sydney, Sydney, NSW Australia; 6 School of Medicine, The University of Western Australia, Crawley, WA Australia; 7 Hawke’s Bay District Health Board, Hastings, New Zealand; 8 Department of Cerebro-Vascular Disease, The People’s 115 Hospital, Ho Chi Minh City, Vietnam; 9 Department of Clinical Neuroscience, Neurology, Karolinska Institutet, Stockholm, Sweden; 10 Center for Safety Research, KTH Royal Institute of Technology, Stockholm, Sweden

**Keywords:** Ischaemic stroke, Haemorrhagic stroke, Antidepressants, SSRI, Fluoxetine, Recovery, Depression

## Abstract

**Background:**

Small trials have suggested that fluoxetine may improve neurological recovery from stroke. FOCUS, AFFINITY and EFFECTS are a family of investigator-led, multicentre, parallel group, randomised, placebo-controlled trials which aim to determine whether the routine administration of fluoxetine (20 mg daily) for six months after an acute stroke improves patients’ functional outcome.

**Methods/Design:**

The core protocol for the three trials has been published (Mead et al., Trials 20:369, 2015). The trials include patients aged 18 years and older with a clinical diagnosis of stroke and persisting focal neurological deficits at randomisation 2–15 days after stroke onset. Patients are randomised centrally via each trials’ web-based randomisation system using a common minimisation algorithm. Patients are allocated fluoxetine 20 mg once daily or matching placebo capsules for six months. The primary outcome measure is the modified Rankin scale (mRS) at six months. Secondary outcomes include: living circumstances; the Stroke Impact Scale; EuroQol (EQ5D-5 L); the vitality subscale of the 36-Item Short Form Health Survey (SF36); diagnosis of depression; adherence to medication; serious adverse events including death and recurrent stroke; and resource use at six and 12 months and the mRS at 12 months.

**Discussion:**

Minor variations have been tailored to the national setting in the UK (FOCUS), Australia, New Zealand and Vietnam (AFFINITY) and Sweden (EFFECTS). Each trial is run and funded independently and will report its own results. A prospectively planned individual patient data meta-analysis of all three trials will provide the most precise estimate of the overall effect and establish whether any effects differ between trials or subgroups. This statistical analysis plan describes the core analyses for all three trials and that for the individual patient data meta-analysis. Recruitment and follow-up in the FOCUS trial is expected to be completed by the end of 2018. AFFINITY and EFFECTS are likely to complete follow-up in 2020.

**Trial registration:**

FOCUS: ISRCTN, ISRCTN83290762. Registered on 23 May 2012. EudraCT, 2011-005616-29. Registered on 3 February 2012.

AFFINITY: Australian New Zealand Clinical Trials Registry, ACTRN12611000774921. Registered on 22 July 2011.

EFFECTS: ISRCTN, ISRCTN13020412. Registered on 19 December 2014. Clinicaltrials.gov, NCT02683213. Registered on 2 February 2016. EudraCT, 2011-006130-16. Registered on 8 August 2014.

**Electronic supplementary material:**

The online version of this article (doi:10.1186/s13063-017-2385-6) contains supplementary material, which is available to authorized users.

## Update

### Introduction

Small trials have suggested that fluoxetine may improve neurological recovery from stroke [1]. FOCUS, AFFINITY and EFFECTS are a family of investigator-led, multicentre, parallel group, randomised, placebo-controlled trials which aim to determine whether the routine administration of fluoxetine (20 mg daily) for six months after an acute stroke improves patients’ functional outcome.

A description of the FOCUS, AFFINITY and EFFECTS core protocol has already been published [1] and that article contained a brief description of planned analyses. However, to avoid any misunderstanding that our final analyses could be post hoc or data-driven, we have published this more detailed statistical analysis plan in advance of completing recruitment. The first draft was prepared by CG and SL before any unblinding to data (see Additional file 1); subsequent drafts were written without input from CG who performed the interim analyses for the FOCUS trial DMC.

Before describing the planned final analyses, the key objectives and methodological features of the three trials are presented.

### Objectives of the individual trials and the prospectively planned individual patient data meta-analysis

We have collaboratively designed and implemented a family of three large, investigator-led, government and charity-funded, multicentre, placebo-controlled randomised trials which together aim to robustly address several research questions.

Our main objective is to determine whether the routine administration of fluoxetine 20 mg daily started 2–15 days post stroke, and continued for six months, improves recovery, and whether any benefits persist after the treatment has stopped, until 12 months after the stroke. The other research questions have been described previously and are included in this analysis plan along with a description of the analyses aimed at addressing each of the questions.

The trials are powered to detect differences in the primary outcome based on an ordinal analysis of the seven categories of the modified Rankin scale (mRS 0, 1, 2, 3, 4, 5, 6) [2]. Because it is not feasible to enrol sufficient patients in each trial to reliably detect small, yet clinically important, effects of fluoxetine, we plan to perform an individual patient data meta-analysis including the data from FOCUS, AFFINITY and EFFECTS.

#### Patient population

Patients are identified by participating clinicians from inpatient stroke services and outpatient clinics in UK (FOCUS), Australasia, New Zealand and Vietnam (AFFINITY) and Sweden (EFFECTS). The full inclusion and exclusion criteria have already been published [1].

#### Randomisation

Having obtained consent, the randomising person enters the baseline data into a trial-specific computerised central randomisation service by means of a secure 24/7 Web interface. After the computer program has checked these baseline data for completeness and internal consistency, it allocates that patient a unique study identification number and a treatment pack number which corresponds to either fluoxetine or placebo. The trial-specific systems apply a common minimisation program to achieve balance for four factors [3]:

- delay since stroke onset (2–8 vs 9–15 days);
- predicted six-month outcome based on the six simple variable (SSV) model [4];
- presence of a motor deficit based on the National Institute of Health Stroke Scale (NIHSS) [5];
- presence of aphasia (based on NIHSS).

The SSV model [4] includes:

1. patient’s age;
2. whether independent in activities of daily living before the stroke;
3. whether living alone before the stroke;
4. whether able to lift both arms off the bed;
5. whether able to walk without the help of another person;
6. whether able to talk, and not confused.

The NIHSS is a 15-item questionnaire where responses to each question are assigned a score in the range of 0–4 (some questions are scored 0–2 or 0–3 as appropriate). The total score is calculated by summing across all questions.

#### Sample size

FOCUS, AFFINITY and EFFECTS are planned to enrol at least 3000, 1600 and 1500 patients, respectively. These numbers would provide 90% power with an alpha of 0.05, based on the distribution of outcomes in the seven categories of the mRS (0–6) observed in both treatment groups combined among the first 451 patients enrolled and followed up at six months in the FOCUS trial. If FOCUS, AFFINITY and EFFECTS combined enrol 6000, we would have 90% power (alpha 5%) to detect a common odds ratio (COR) of 1.16, equivalent to a 3.7% absolute difference in percentage with mRS 0–2 (44.0% to 47.7%). The trial steering committees (TSC) will regularly review the target sample size and adjust this based on accruing blinded data.

#### Blinding

The patient, their families, the healthcare team—including the pharmacist—and anyone involved in outpatient assessments are blinded to the treatment allocation. Emergency unblinding systems are available for each trial.

#### Follow-up

Early follow-up, during the index admission and in the first month to identify adverse events and monitor adherence is carried out by the local centres in all three trials. However, each trial’s national coordinating centres aim to follow-up the patients at about six and 12 months with postal and telephone questionnaires to measure the primary and secondary outcomes. However, the trials vary in the timing, frequency and method of monitoring the patients’ progress. Data are also collected from general practitioners and by data linkage to mortality and hospital admission data in all three trials. The reasons why patients stopped taking the trial medication are recorded.

#### Primary outcome

The primary outcome is functional status, measured with the mRS [2] at the six-month follow-up. We are using the simple modified Rankin scale questionnaire (smRSq) delivered by postal questionnaire or via interview over the telephone or face-to-face to determine the mRS [6–8]. The smRSq is based on the yes/no responses to five questions [7].

#### Secondary outcomes

1. Survival till the end of the trial. This is determined by following patients up for 12 months through their GPs and telephone and postal questionnaire and thereafter through linkage to routine mortality data.
2. Functional status (mRS) at 12-month follow-up.
3. Health status with the Stroke Impact Scale (SIS) comprising 59 questions divided into nine domains on each of which the patient scores 0–100 [9–11]. The domains cover:
  - Arm, hand, leg and foot strength;
  - Hand function;
  - Mobility;
  - Daily activities;
  - Communication and understanding;
  - Memory and thinking;
  - Mood and emotions;
  - Participation in work, leisure and social activities;
  - Recovery rated on a vertical visual analogue scale (VAS; 0–100).

The domain scores are derived using the formula [9]:

$$ \mathrm{Transformed}\kern0.17em \mathrm{scale}=\left[\left(\mathrm{raw}\;\mathrm{score}\hbox{--} \mathrm{lowest}\kern0.17em \mathrm{possible}\;\mathrm{raw}\;\mathrm{score}\right)/\mathrm{possible}\;\mathrm{raw}\;\mathrm{score}\kern0.17em \mathrm{range}\right]\ast 100 $$

4. Adverse events/outcomes:
  - depression. A new diagnosis and/or treatment of depression during follow-up. Participants (or a proxy or GP) are asked if they have been diagnosed with new depression since their last assessment, whether this has been treated and whether they have been started on an antidepressant medication;
  - recurrent stroke including ischaemic and haemorrhagic strokes;
  - acute coronary syndromes;
  - epileptic seizures;
  - episodes of hyponatraemia (< 125 mmol/L);
  - upper gastrointestinal (GI) bleeding;
  - other major bleeds (lower GI, extracranial, intracranial but extracerebral);
  - poorly controlled diabetes including hyperglycaemia (> 22 mmol/L) and symptomatic hypoglycaemia;
  - falls resulting in injury;
  - new bone fractures;
  - attempted suicide/self-harm.
5. Fatigue (Vitality subscale of the 36-Item Short Form Health Survey [SF36]) [12, 13]. In the full SF36, the vitality questions are 9a, 9e, 9 g, 9i. For a and e, these are scored 5 points for ‘all of the time’ through to 1 point for ‘none of the time’ while g and i are scored 1 point for ‘all of the time’ through to 5 points for ‘none of the time’. A raw score is then calculated by summing across the four questions, resulting in a score of 4–20 with a range of 16. The raw scores are then transformed to a score in the range of 0–100 using the formula:
  Transformed scale = [(raw score – lowest possible raw score)/possible raw score range] * 100 [14].
6. Cognition. The SIS which incorporates an assessment of memory and thinking is used for all three trials [9–11]. In AFFINITY, cognition during follow-up is assessed with the Modified Telephone Interview for Cognitive Status (TICSm) [15]. EFFECTS assesses cognition with the Montreal Cognitive Assessment (MoCA) at baseline and six months (face-to-face) [16]. Only the analysis of the SIS is described.
7. Mood. The individual trials used different scales to compare the mood of patients at follow-up using: Mental Health Inventory 5 (MHI) in FOCUS [17]; PHQ9 in AFFINITY [18]; and MADRS in EFFECTS [19].
8. Health-related quality of life measured with the five-level Euroqol (EQ5D-5L) to generate utilities based on population preferences [20, 21].

The EQ5D-5L consists of five dimensions, each with five levels of perceived problems: Level 1 = no problem; Level 2 = slight problems; Level 3 = moderate problems; Level 4 = severe problems; Level 5 = extreme problems/unable to perform. Each level is assigned a numerical code (level 1 = code 1, level 2 = code 2, etc.). These dimensions are combined to give a five-digit code with any missing dimension coded as ‘9’. Each health state is assigned a single index-based numerical value (‘utility’) that can be combined with survival times to generate quality-adjusted life years (QALYs). Initially we will use the largest and most recent EQ-5D-5 L value set calibrated for an English population [21] but will apply corresponding population specific value sets as they appear for the centres/countries participating in this family of trials (https://euroqol.org/eq-5d-instruments/eq-5d-5l-about/).

Each trial is collecting data about resource use over the first 12 months to enable us to carry out health economic analyses; these will be trial-specific but a combined analysis will be undertaken including data common to all three trials.

#### Statistical analysis plan

We will present a CONSORT diagram and a table of baseline data (see Table 1) but will not carry out any testing of statistical significance of difference between treatment groups unless required by the publishing journal [22]. For all analyses, unless otherwise specified, we will retain participants in the treatment group to which they were originally assigned, irrespective of the treatment they actually received (i.e. an intention-to-treat analysis).

**Table 1:** Baseline data collected before randomisation in the three trials

Data item	Fluoxetine	Placebo
Age (years (mean, SD))		
Gender (male %)		
Ethnicity		
Marital status		
Living arrangements		
Employment		
Independent in everyday activities before the stroke		
Patient consented for themselves		
Co-morbidities (existing)		
Previous ischaemic stroke/TIA		
Previous intracranial bleeding		
Known coronary heart disease		
Current depression		
Past depression		
Known diabetes mellitus		
Previous upper gastrointestinal bleeding		
Current or past hyponatraemia (Na < 130 mmol/L)		
Previous bone fractures		
Status at enrolment		
NIHSS (median, IQR)		
Motor deficit present on NIHSS		
Aphasia present on NIHSS		
Ability to lift both arms		
Ability to walk alone		
Able to talk and not confused (GCS speech normal)		
Patient health questionnaire (PHQ)		
Haemorrhage on brain imaging?		
OCSP classification for ischaemic stroke		
Modified TOAST classification		

The final analyses will be performed after the final patient has been recruited and followed up for a minimum of 12 months post randomisation. The final analyses will be performed on the dataset after any ‘cleaning’ that may be required has been completed and the database locked. The treatment allocation will only then be unblinded.

The primary analysis will be programmed by CG and also independently by a second statistician and the results will be compared; any inconsistencies will be identified and resolved by discussion. The rest of the analysis report will be scrutinised for unexpected findings and checked for accuracy.

A statistical significance level of *p* < 0.05 (two-tailed) will be applied to all analyses. All adjusted analyses will be adjusted for the variables included in our minimisation algorithm with presence of motor deficit and aphasia as binary variables as defined above but including delay since stroke onset (days) and predicted probability of a good outcome at six months as continuous variables.

### Primary analysis

This aims to address our primary research question: does the routine administration of fluoxetine (20 mg o.d.) for six months after an acute stroke improve patients’ functional status at six months?

To minimise missing data, our analyses of the primary outcome will include a mRS obtained between 90 days and one year after randomisation, taking the value measured closest to the six-month time point. The distribution of delays from randomisation to this follow-up will be presented to ensure it does not differ between treatment groups.

We will present the number and percent of patients in each treatment group by mRS category. In our comparison of mRS at six months post randomisation by treatment group, the mRS will be treated in its full ordinal format (scores of 0, 1, 2, 3, 4, 5, 6) [23]. This will be examined using an ordinal logistic regression and will be performed in both an unadjusted manner and also adjusted for factors in the baseline minimisation. The latter will be our primary estimate of treatment effect as recommended by the Medicines and Healthcare products Regulatory Agency (UK). We will present common odds ratios (ORs) with 95% confidence intervals (CI) using the proportional odds method and for the unadjusted analysis we will also present the individual dichotomies. We will calculate absolute reductions in risk from these values along with 95% CIs. We will give a *p* value. In addition, the results will be presented as relative risks with 95% CI, calculated from the OR [24] and absolute risk differences with 95% CI.

#### Secondary analyses

Analyses of secondary outcomes and analysis of our primary outcome (ordinal mRS) in pre-defined subgroups will be performed to address the other research questions listed in our protocol. Where the outcome of interest is binary, comparison by treatment group will be examined using a binary logistic regression and will be adjusted for factors used in the minimisation algorithm. These will be presented as ORs and 95% CIs, absolute risk reductions with 95% CI and relative risks using the same method as for the primary outcome. Unadjusted ORs and 95% CIs will also be presented.

Where the outcome of interest is continuous, descriptive statistics will be presented (N, mean, sd, min, max, median, Q1, Q3) categorised by allocated treatment. Due to the nature of the distribution of these measures in this population, a simple unadjusted analysis will be performed comparing the two treatment groups using a Mann–Whitney test (i.e. not adjusted for variables in the minimisation algorithm). If the data are not of the required form (for instance if ≥ 75% of respondents have the same value), we will use another suitable method.

This analysis will be conducted for the following outcomes at both six and 12 months:

1. Fatigue measured by the vitality sub-scale of the SF36.
2. Individual SIS Domain scores, a ‘Motor score’ derived from averaging scores across three domains (Arm, hand, leg and foot strength; Hand function; and Mobility), a ‘Physical function score’ derived by averaging across four domains (Arm, hand, leg and foot strength; Hand function; Mobility; and Daily activities) and Recovery based on the VAS.
3. Cognition will be compared between the placebo and fluoxetine groups using the SIS domain Memory and thinking in all trials, and in individual trials using the TICs-M (AFFINITY) and MOCA (EFFECTS).
4. Mood will be compared between the placebo and fluoxetine groups in all trials with the Mood and emotions domain of the SIS, and in individual trials using MHI [17] in FOCUS, PHQ9 [18] in AFFINITY and MADRS [19] in EFFECTS.
5. Quality of life as measured by the EQ5D-5L.

Our analyses aim to answer the following questions:

1. If fluoxetine improves functional status (mRS) at six months, does any improvement in functional status persist after treatment is stopped? To answer this question, we will use ordinal regression to compare functional status (mRS scores) at the 12-month follow-up, as for our primary analysis.
2. Does fluoxetine influence the secondary outcome measures (living circumstances, quality of life, fatigue, stroke impact and mood) at six months and/or 12 months? The binary outcomes are: living at home or with relative vs care home; hospital or long-term care; mRS at six months and 12 months (mRS 0–2 vs mRS 3–6); new diagnosis of depression corroborated by the GP or hospital after randomisation by six months and 12 months. The continuous outcomes are EQ5D-5L, Vitality subscale of SF36, SIS, MHI5, PHQ9 and MADRS.
3. Does fluoxetine increase the risk of serious adverse events? We will compare the proportion of patients having any of the following adverse events (all binary outcomes) between randomisation and cessation of the trial medication (i.e. investigational medicinal product), based on treatment received rather than intention to treat:
  - any recurrent stroke;
  - ischaemic stroke (not TIAs);
  - haemorrhagic stroke;
  - acute coronary syndromes;
  - epileptic seizure;
  - episode of hyponatraemia (< 125 mmol/L);
  - upper gastrointestinal bleeding;
  - other major bleeds (lower GI, extracranial, intracranial but extracerebral);
  - poorly controlled diabetes including hyperglycaemia (> 22 mmol/L) or symptomatic hypoglycaemia;
  - falls resulting in injury;
  - new fractures;
  - attempted suicide/self-harm.
4. If fluoxetine is effective, is it also cost-effective? Each trial has taken slightly different approaches to the estimating the cost-effectiveness of the interventions. These have been tailored to their national circumstances. We envisage performing an individual patient data meta-analysis to derive the best estimate of overall cost-effectiveness and that in subgroups of patients. At this stage when it is unclear which data will be available in which trials, we cannot be too specific about the statistical methods to be used.
  We plan to carry out within-trial economic analysis of direct resource costs and health outcomes on an intention-to-treat basis. A health service perspective will be adopted for measuring and valuing health service use over a 12-month time horizon, although some data to reflect indirect costs will be available in the EFFECTS trial (Sweden).
  We will estimate cumulative costs of inpatient episodes, hospital outpatient visits, care home stays and home care visits. Unit costs will be obtained from each participating country (Australia, New Zealand and Vietnam, Sweden and the United Kingdom) and applied to patient-level data collected within each international setting.
  The number and duration of hospital episodes and other secondary care contacts will be recorded using linkage to routine data or information obtained from the case report form. Resource use will be valued using unit costs taken from national datasets and published sources (e.g. Australian National Hospital Cost Data Collection, UK Unit Costs of Health and Social Care, Swedish National Cost per Patient register). Unit prices will be applied to resource use data using 2018 as the reference year with country-specific health-sector price deflators used to adjust costs reported in other years. No discounting of direct resource costs will be conducted as the time horizon will be limited to 12 months for within-trial analysis. Output-based hospital-specific purchasing power parities (PPPs) will be used for conversion of expenditure estimates using a common (currency) unit.
  Self-reported health-related quality of life (HRQoL) at six and 12 months of follow-up will be measured using the EQ-5D-5 L preference based scale. EQ-5D-5 L single index values will be calculated using English value sets updated if value sets are reported for other countries where the family of trials are being conducted. We also plan to validate the EQ-5D-5 L by checking the concordance with the mRS.
  A standard multiplicative model will be used to estimate QALYs calculated by the area under linear interpolation of the EQ-5D-5 L index trajectory for each individual with survival times, the EQ-5D-5 L utility index score at six and 12 months, and a modelled baseline EQ-5D-5 L utility index value. Multiple imputation with chained equations will be used to impute missing HRQoL data on the EQ-5D-5 L assuming missing at random [25]. We will perform sensitivity analysis based on cases with complete data follow-up and examine whether the pattern of missingness is informative with respect to key individual characteristics.
  The primary treatment effect in the economic analysis will be estimated using an individual level regression model for average (mean) incremental costs and incremental QALY times over 12 months after randomisation. The model will consider the joint distribution of costs and QALYs using a general specification that will allow for different parametric and conditional distributions. We also plan to use a Bayesian model with minimally informative priors for means and large variances [26]. Model parameter uncertainty will be addressed using probabilistic sensitivity analysis summarised using the cost-effectiveness acceptability curve. We will also conduct a companion analysis of cost-effectiveness where we will truncate the cumulative cost distribution at six months and estimate the incremental costs in relation to incremental differences in the primary outcome measure (mRS at six months).
  Secondary analyses will be conducted to address heterogeneous treatment effects. Subpopulations with different average treatment effects will be identified using ‘regression tree’ or ‘recursive partitioning’ methods [27]. These data-driven analyses will complement pre-specified subgroup analyses examining individual and group covariates of substantive interest such as stroke severity (NIHSS) and the SSV model for prognosis [4].
  Longer run modelling will estimate the distribution of costs and QALYs calculated over the expected patient lifetimes. A microsimulation model will be calibrated using information gained from the within trial analysis of cost-effectiveness combined with additional data from: (1) trials and observational studies reporting longer run costs, survival and HRQoL following stroke; and (2) expert beliefs on the distributions of parameters where information is less readily available. The structural uncertainty in the long run model will be addressed using model averaging methods.
  The design of the economic analysis will contribute to a structured overview of treatment effects taking advantage of the common trial protocols and consistent capture of resource use and outcomes in FOCUS, AFFINITY and EFFECTS. Generalisability and, in particular, the assessment of treatment effect heterogeneity will be enhanced by the pooled data across these trials.
5. Is fluoxetine associated with longer-term survival? Functional outcome at six months post stroke is strongly associated with long-term survival and so we wish to determine whether any benefits on functional outcome would translate into longer-term survival [28]. We will use Cox proportional hazards regression to analyse the effect of treatment on survival to 12 months. We will adjust for the variables included in our minimisation algorithm. We will present this analysis graphically (cumulative hazard of death [%] vs time), providing a hazard ratio with 95% CIs and a *p* value. This analysis will be repeated if survival data for a more prolonged period becomes available and sufficient resources are available to perform and report the analyses.
6. Does the presence or absence of any of the following factors materially alter the effect of fluoxetine on our primary outcome:
  - stroke pathology (ischaemic vs haemorrhagic vs uncertain pathological type);
  - age (≤ 70 years, > 70 years);
  - stroke severity, i.e. baseline probability of a good outcome on mRS calculated with the SSV model [4] to see if effects remain constant across the range of stroke severities (< 0.15 vs 0.15–1);
  - patients who were unable to consent for themselves, since this subgroup will allow us to address the question whether routine use of fluoxetine is likely to benefit patients in whom a formal assessment of mood is impossible because of communication and cognitive problems;
  - inability to assess mood because of communication or cognitive problems (NIHSS Q1b > 0 or Q1c > 0 or Q9 > 1 or Unable to answer PHQ2 [29] at randomisation). Defining the patients’ ability to have their baseline mood assessed based on NIHSS and PHQ is likely to be more meaningful than based on patient or proxy consent (see d above), especially since no proxy consent is allowed in EFFECTS (Sweden);
  - patients with and without depression at baseline. Depression at baseline is defined as ‘Current depression at baseline’ or answers yes to both questions in PHQ2, because the systematic review [30] suggested that the effect of selective serotonin reuptake inhibitors (SSRI) was greater in those who were depressed.
  The functional status (mRS) at six months will be compared with ordinal regression in these mutually exclusive subgroups by entering a treatment by subgroup interaction into the regression model.
7. In patients with motor deficits at randomisation, does fluoxetine improve motor function? Patients with a motor deficit affecting the face/arm or leg (based NIHSS Q5, 6, 7, 8, 9 > 0). For this subgroup analysis in addition to comparing their overall functional outcome based on the ordinal analysis of mRS, we will compare with Motor score, and Physical function scores based on the SIS domains described above.
8. In patients with aphasia at randomisation, does fluoxetine improve communication? Patients with aphasia (based on NIHSS Q9 > 0). For this subgroup, analysis in addition to comparing their overall functional outcome based on mRS based on ordinal analysis we will compare with SIS – communication subscale.
  For questions 7 and 8, because patients may have a combination of neurological deficits, individual patients may appear in more than one subgroup.
9. Is there a relationship between functional status at six months and mood and is this relationship affected by fluoxetine? We will perform exploratory analyses of potential mediating factors, e.g. the role of depression. These will not be reported in the primary publication. We will seek to answer the question whether any benefits are mediated by improvement in mood (based on MHI5 in FOCUS, PHQ9 in AFFINITY and MADRS in EFFECTS) and also whether any apparent loss of benefits in mRS or SIS at 6–12 months is because of a deterioration in mood.

### Missing data

Our randomisation systems do not allow investigators to proceed to treatment allocation without entering complete baseline data (Table 1). The mRS, our primary outcome, includes death, so it is expected that the number with missing mRS at follow-up will be minimal. Anyone with missing mRS will not be included in any analysis requiring mRS (complete case analysis).

For secondary outcomes where missing data are expected because data will not be available for patients who have not survived, we will present results for those who are alive at follow-up and any discrepancy in death rates between groups will be taken into account in the interpretation. Missing data for single questions within scores will be handled as detailed by each scoring method, where responses to all questions within a scale or subscale are missing that patient will not be included in that part of the analysis. Immediately before database lock, we will carry out a blinded review of the completeness of primary and secondary outcomes. If we see higher levels of missing mRS data than expected (> 5%), we will use a suitable analysis, based on the likely missing data mechanism. We will consider whether to extend missing data methods to secondary outcomes. If required due to number of patients with missing scores, a sensitivity analysis may be performed.

### Protocol deviations, adherence and blinding

Inclusion/Exclusion violations: we will report the number and percentage of participants randomised who did not meet the entry criteria, e.g. non-strokes, with exclusion criteria. However, they will be included in the primary analysis. A secondary analysis will exclude ineligible patients (see below).

Unblinding: We will report the number of patients who required unbinding of study medication during the trial by treatment arm and where available present the reasons for unblinding.

Adherence: Each participant is issued with a total of six months’ supply of trial medication. At six months, they are asked if they have completed the course and taken all the capsules and also on average how often they took capsules. They are asked the reasons for stopping, as well as the date of stopping. Where possible, we retrieve and count the unused trial medication. Before unblinding we will derive an estimated date on which the patient was thought to have taken their last dose of trial medication and use the interval (days) from first dose to that date as our main measure of adherence. This will be based on all of the available information. We will use a combination of:

- inclusion/exclusion violations;
- the answers to the adherence questions (see above);
- number, percentage and duration of any open label SSRI intake before the six-month follow-up;
- the reasons for stopping trial medication

to define several types of non-adherence to the protocol (see 1-8 below).

A so-called intention-to-treat analysis, where the outcomes of patients are analysed in the groups they were randomised to regardless of treatment received, provides the least biased and most robust evidence of the effect of treatment. However, the observed treatment effect may be reduced if a large number of patients are included who are unlikely to benefit because they did not have a stroke or more likely where a large proportion of patients do not receive the allocated treatment or actually received the alternative treatment (i.e. cross-overs).

If the primary analysis in the individual trials or the individual patient data meta-analysis does not demonstrate an improvement of functional outcome (mRS) at the six-month follow-up, the question will arise whether this is this likely to be due to poor adherence to the protocol and/or trial medication? This is important since we would not wish to abandon a potentially useful treatment simply because of poor adherence to trial protocols or trial medication. These might be improved in any future trials. We will perform further per protocol analyses to reassure the clinical community that the trials have not underestimated any treatment effect to an extent which would alter future clinical practice or, more likely, the need for further randomised trials of SSRIs in stroke.

Inevitably, analyses which try to take account of adherence introduce a degree of patient selection and thus are likely to introduce bias.

These exploratory sensitivity analyses to account for non-adherence will include all of the analyses of the primary outcome and selected secondary outcomes:

- living at home or with relatives vs care home, hospital or long-term hospital care;
- mRS at six months and 12 months (mRS 0–2 vs mRS 3–6);
- new diagnosis of depression since randomisation by six months and 12 months;
- SIS domain scores;
- averaged score over all SIS domains;
- SF36 vitality subscale score;
- utility based on EQ5D-5L and population preferences.

These analyses will not include any analysis of subgroups defined on the basis of baseline variables. The following groups will be sequentially added to the group excluded from the analyses:

1. Patients who did not meet the entry critria for the trial.
2. Patients who did not receive any trial medication.
3. Patients who received < 90 days of trial medication because of failures in trial procedures, e.g. failures to transfer trial medication with patients during moves between hospitals, care homes and home. The 90-day cut-off was chosen because previous trials have tested this duration of treatment with apparent benefit [30].
4. Patients who received trial medication for < 90 days because of patient or relative concerns but not due to suspected adverse reactions.
5. Patients who received < 90 days of trial medication because they experienced symptoms which were attributed to the trial medication.
6. Patients who had been allocated to placebo who received an SSRI (fluoxetine or other) within the first 90 days and which was not known to have been stopped within ten days of starting.
7. Patients who had been allocated to fluxoetine who received an SSRI (fluoxetine or other) within the first 90 days and which was not known to have been stopped within ten days of starting.
8. Patients who did not complete at least 150 days of treatment. We chose this cut-off because patients sometimes received the questionnaires shortly before six months and some stopped the trial medication at that point, while others finished off the 186 capsules. We regarded both as fully adherent.

#### Individual patient data meta-analysis

This will allow us: to provide the most precise estimates of any risks and benefits; to detect reliably a smaller overall effect size than those detectable by the individual trials; to better determine the effects of fluoxetine vs placebo in subgroups; and to broaden the generalisability of the results. It also provides the best opportunity to determine whether any combinations of baseline characteristics might be associated with larger treatment effects. Also, it aims to identify where the treatment effect differs between studies since we hypothesise that characteristics of the healthcare systems in which the trials were performed and the trial processes which were tailored to the local healthcare systems may influence the treatment effect. For instance:

- the method of dispensing, prescribing and optimising adherence to the trial medication differs between the trials;
- the location, intensity and duration of rehabilitation differs between healthcare systems;
- whether patients with depression were included in the study (as in FOCUS and AFFINITY) or not (as in EFFECTS).

Before performing analyses of estimated treatment effects (see below), we will perform descriptive and exploratory analyses to identify and display differences in baseline characteristics between the types of patient enrolled in the three trials; specifically, statistical comparisons of baseline means (using t-tests) and prevalence (using chi-squared tests) between patients enrolled in the three trials. Also, we will describe the duration and type of hospital stays between randomisation and discharge home, discharge to a residential or nursing home, or death. The reason for carrying out these initial analyses is to aid the interpretation of any apparent between-trial treatment differences that may be identified.

The individual patient data meta-analysis will include all of the analyses described for the individual trials, including the same subgroup analyses, on the combined dataset. All regression models (linear, logistic, Cox proportional hazards) will be meta-analysed across trials using a ‘two-stage’ approach. ‘The first stage will be to perform the ordinal logistic, logistic and Cox proportional hazards regressions separately for each trial, as described for the main within-trial analysis. The second stage will take the point estimate and standard error of the treatment effect from the within-trial analyses. These will be meta-analysed using a generic inverse variance model. Fixed effect analyses will be performed and presented.

#### Trials status

FOCUS recruited its first patient on 10 September 2012 and its last on 31 March 2017, AFFINITY recruited its first patient on 11 January 2013 and EFFECTS on 20 October 2014. Our target is to complete recruitment and follow-up in all three trials by 2020.

## Additional file

Additional file 1: The draft statisitcal analysis plan written by CG and SL prior to any unblinding to interim analyses of FOCUS trial. (DOC 978 kb)

## Abbreviations

AFFINITY: Assessment oF FluoxetINe In sTroke recoverY
CI: Confidence intervals
COR: Common odds ratio
CTIMP: Clinical Trial of an Investigational Medical Product
DMC: Data monitoring committee
DSM IV: Diagnostic and Statistical Manual of Mental Disorders Version IV
EFFECTS: Efficacy oF Fluoxetine – a randomisEd Controlled Trial in Stroke
eGFR: Estimated glomerular filtration rate
EQ5D-5L: EuroQol 5 dimension questionnaire – five level
FOCUS: Fluoxetine Or Control Under Supervision
GI: Gastrointestinal
MADRS: Montgomery-Åsberg Depression Rating Scale
MAOI: Monoamine oxidase inhibitor
MHI5: Mental Health Inventory 5 questions
MoCA: Montreal Cognitive Assessment
mRS: Modified Rankin scale
NIHSS: National Institutes of Health Stroke Scale
OCSP: Oxfordshire Community Stroke Project
PHQ: Patient health questionnaire
PPP: Purchasing power parities
RR: Relative risk
SF36: 36-Item Short Form Health Survey
SIS: Stroke Impact Scale
smRSq: Simple modified Rankin scale questionnaire
SSRI: Selective serotonin reuptake inhibitor
TICSm: Modified Telephone Interview for Cognitive Status
TOAST: Trial of ORG 10172 in Acute Stroke Treatment
UK: United Kingdom
