# Supplementary material for: The FOCUS, AFFINITY and EFFECTS trials studying the effect(s) of fluoxetine in patients with a recent stroke: statistical and health economic analysis plan for the trials and for the individual patient data meta-analysis
Source: Trials. 2017 Dec 28;18:627. doi: 10.1186/s13063-017-2385-6 (PMC5745973; doi:10.1186/s13063-017-2385-6)
Supplement: Additional file 1: — The draft statisitcal analysis plan written by CG and SL prior to any unblinding to interim analyses of FOCUS trial. (DOC 978 kb) [file 13063_2017_2385_MOESM1_ESM.doc]

| Trial Title | **Fluoxetine Or Control Under Supervision [FOCUS]** |
| --- | --- |
| EudraCT Number | **2011-0056 16-29** |
| REC Number | **11/SS/0100** |
| Chief Investigators | **Dr Gillian Mead**  Department of Geriatric Medicine 2nd Floor  Royal Infirmary of Edinburgh  Little France, Edinburgh, EH16 4TJ  Tel: 0131 242 6369  Email: Gillian.e.mead@ed.ac.uk  **Professor Martin Dennis**  Neurosciences Trials Unit  Bramwell Dott Building, Western General Hospital  Edinburgh, EH4 2XU  Tel: 0131 537 1719, Mob: 07979708008  Email: martin.dennis@ed.ac.uk |
|  | |
| Document | **Statistical Analysis Plan [SAP]** |
| Version number, date | **1, 03 December 2013** |
| Based on protocol version, date | **Version 3 16/05/2012** |
| Author | **C.Graham [Trial statistician]** |
|  | |
| Approved by | Dr G. Mead: ________________ Date: __________  Prof M. Dennis: ________________ Date: __________  Dr S. Lewis: ________________ Date: __________ |

**INDEX**

[1. List of abbreviations 4](#__RefHeading___Toc374433233)

[2. Introduction 5](#__RefHeading___Toc374433234)

[3. Statistical methods from protocol 5](#__RefHeading___Toc374433235)

[4. Study Methods 6](#__RefHeading___Toc374433236)

[4.1. Randomisation and blinding 6](#__RefHeading___Toc374433237)

[4.2. Study Assessments 7](#__RefHeading___Toc374433238)

[4.3.1. Stroke Impact Scale [SIS] 8](#__RefHeading___Toc374433239)

[4.3.2. Mental Health Inventory 5 [MHI-5] 8](#__RefHeading___Toc374433240)

[4.3.3. EuroQol 5D-5L [EQ5D-5L] 8](#__RefHeading___Toc374433241)

[4.3.4. Short Form 36 [SF36] Vitality 9](#__RefHeading___Toc374433242)

[4.3.5. National Institute of Health Stroke Score [NIHSS] 9](#__RefHeading___Toc374433243)

[4.3.6. Six Simple variable (SSV) model 9](#__RefHeading___Toc374433244)

[4.3.7. Modified Rankin Score [mRS] 10](#__RefHeading___Toc374433245)

[5. General Considerations 11](#__RefHeading___Toc374433246)

[5.1. Overall 11](#__RefHeading___Toc374433247)

[5.1.1. Timing of Final Analysis 11](#__RefHeading___Toc374433248)

[5.1.3. Missing Data 11](#__RefHeading___Toc374433249)

[5.2. Interim Analysis 11](#__RefHeading___Toc374433250)

[5.2.1 Interim Analysis, Data Monitoring and Stopping Rules 11](#__RefHeading___Toc374433251)

[5.2.2 Interim Analysis and Sample Size Adjustment 12](#__RefHeading___Toc374433252)

[5.2.3. Practical Measures to Minimise Bias 12](#__RefHeading___Toc374433253)

[5.2.4. Other evidence 13](#__RefHeading___Toc374433254)

[6. Summary of DMC Report Data 13](#__RefHeading___Toc374433255)

[6.1. Recruitment 13](#__RefHeading___Toc374433256)

[6.2. Trial randomisation flow chart 13](#__RefHeading___Toc374433257)

[6.3. Success of minimisation, and baseline data 13](#__RefHeading___Toc374433258)

[6.4. Protocol violations, compliance and blinding 14](#__RefHeading___Toc374433259)

[6.5. Adverse events 14](#__RefHeading___Toc374433260)

[6.6. Outcomes 14](#__RefHeading___Toc374433261)

[7. Final analysis 15](#__RefHeading___Toc374433262)

[7.1. Primary outcome 15](#__RefHeading___Toc374433263)

[7.2. Secondary outcomes - binary 15](#__RefHeading___Toc374433264)

[7.3. Secondary outcomes - ordinal 15](#__RefHeading___Toc374433265)

[7.4. Secondary outcomes - continuous 15](#__RefHeading___Toc374433266)

[7.5. Secondary outcomes – survival 16](#__RefHeading___Toc374433267)

[7.6. Pre-specified sub group analysis 17](#__RefHeading___Toc374433268)

[7.7. Possible extra analyses 17](#__RefHeading___Toc374433269)

[8. Revision History 18](#__RefHeading___Toc374433270)

## 1. List of abbreviations

| CHD | Chronic Heart Disease |
| --- | --- |
| CI | Confidence Interval |
| DMC | Data Monitoring Committee |
| EQ5D-5L | EuroQol 5D -5 Long |
| FOCUS | Fluoxetine Or Control Under Supervision |
| GI | Gastrointestinal |
| HRQOL | Health Related Quality of Life |
| MHI5 | Mental Health Inventory 5 |
| mRS | Modified Rankin Score |
| NIHSS | National Institute of Health Stroke Score |
| OCSP | Oxford Community Stroke Project |
| SF-36 | Short Form-36 |
| SIS | Stroke Impact Scale |
| SSV | Six Simple variable |
| TIA | Transient Ischaemic Attack |
| TSC | Trial Steering Committee |
|  |  |
|  |  |

## 2. Introduction

Fluoxetine Or Control Under Supervision (FOCUS) is a parallel-group multicentre randomised trial to establish the effect(s) of routine administration of Fluoxetine in patients with a recent stroke. The trial aims to recruit at least 3000 patients. Patients are randomised via a web based randomisation system utilising a minimisation algorithm with a random component to receive Fluoxetine 20mg once daily or matching placebo capsules for 6 months and follow up at 6 and 12 months.

## 3. Statistical methods from protocol

Our primary analyses will retain patients in their original assigned treatment groups.

Our primary analysis will compare the mRS at the six month follow up using an ordinal analysis adjusted for any baseline imbalance in those factors included in our minimisation algorithm.

We will compare the mRS at the twelve month follow up to establish if any benefits observed at 6 months are maintained.

Secondary analyses will compare the two treatment groups with respect to the following outcomes at 6 and 12 months.

- Survival (Logistic regression)
- EQ5D-5L (HRQOL) to generate utilities
- SIS (for each of 9 domains on which the patient scored 0-100)
- MHI 5 (mood)
- Fatigue (Vitality subscale of SF36)
- New diagnosis of depression requiring treatment with antidepressants
- Adverse events
- Adherence to trial medication

Longer term survival will be analysed with Cox proportional hazards model

We will also perform analyses of potential mediating factors e.g. the role of depression. We will seek to answer the question whether any benefits are mediated by improvement in mood (based on MHI 5 and also whether any apparent loss of benefits in mRS or SIS between 6 months to 12 months is because of a deterioration in mood.

**Pre-defined subgroups**

The mRS will be compared at six months with an ordinal analysis in the following subgroups:

- Age (≤70, > 70yrs)
- Baseline probability of a good outcome on mRS (Counsell 2002) – to see if effects remain constant across the range of stroke severities (<0.15 vs 0.15-1 probability of being alive and independent at 6 months)
- Ischaemic vs haemorrhagic stroke
- Patient who were unable to consent for themselves since this subgroup will allow us to answer the question whether routine use of fluoxetine is likely to benefit patient in whom a formal assessment of mood is impossible because of communication and cognitive problems.

In addition we are particularly interested to know whether the effect of treatment on neurological function is modified by specific neurological deficits present at baseline. Because patients may have a combination of neurological deficits, individual patients may appear in more than one subgroup

Patients with a motor deficit (i.e. weakness or clumsiness on NIHSSS) affecting face, arm or leg.

- Relevant outcomes – SIS – Strength, mobility, hand/arm function
- Patients with aphasia based on the NIHSS
- Relevant outcomes – SIS - communication

## 4. Study Methods

### 4.1. Randomisation and blinding

Having obtained consent, the randomising person collects the baseline data necessary to complete a randomisation form and enters the patient’s baseline data into our computerized central randomisation service by means of a secure 24/7 Web interface or a telephone call to the trial office during office hours. After the computer program has checked these baseline data for completeness and consistency it allocates that patient a unique study identification number and a treatment pack number which corresponds to either Fluoxetine or Placebo. The system applies a minimisation program to achieve balance for four factors:

- Delay since stroke onset [2 – 8 days v 9 – 15 days]
- Predicted outcome at 6 months based on Counsell 2002 [predicted probability of being alive & independent at 6 months of 0 – 0.15 v 0.16 – 1]
- Presence of a motor deficit [yes v no – where deficit is NIHSS5 (right or left) >0, NIHSS6 (right or left)>0 or NIHSS7 >0]
- Presence of aphasia [NIHSS question 9: 0 v 1 – 3 ]

The minimisation algorithm randomly allocates the first patient to a treatment, but allocates each subsequent patient to the treatment that leads to the least difference between the treatment groups with respect to the prognostic factors (Altman 2005). To ensure that we retain a random element to treatment allocation, patients will be allocated to the group which minimizes differences between groups with a probability of 0.8. The system contains a list of treatment codes for each centre and which match the stocks held at that centre. At the end of the session each patient is allocated a treatment code which corresponds to either an active (f.luoxetine 20mg once daily) or placebo treatment pack which contains six months supply of capsules held at that centre. The randomisation system will take account of the drug stocks held locally to firstly ensure the allocated treatment is available and second to minimise wastage.

The patient, their families, the healthcare team including the pharmacist and anyone involved in outpatient assessments will be blinded to the treatment allocation.

### 4.2. Study Assessments

|  | Days | Weeks | | | | | | | |
| --- | --- | --- | --- | --- | --- | --- | --- | --- | --- |
| **Assessment** | 2-15 | 4-6 | 12 | 24 | 26 | 30 | 50 | 52 | 54 |
| ***Local*** |  |  |  |  |  |  |  |  |  |
| Screen of eligibility | x |  |  |  |  |  |  |  |  |
| Check results of post stroke bloods | x |  |  |  |  |  |  |  |  |
| Give PIB to patient and/or carer | x |  |  |  |  |  |  |  |  |
| CONSENT | x |  |  |  |  |  |  |  |  |
| Collect baseline data | x |  |  |  |  |  |  |  |  |
| Randomise | x |  |  |  |  |  |  |  |  |
| Record treatment code/study no. | x |  |  |  |  |  |  |  |  |
| Prescribe study medication | x |  |  |  |  |  |  |  |  |
| Dispense 6 months of treatment | x |  |  |  |  |  |  |  |  |
| FAX no. of dispensed medication | x |  |  |  |  |  |  |  |  |
| Complete discharge form including |  | + |  |  |  |  |  |  |  |
| Adverse events |  | + |  |  |  |  |  |  |  |
| All medications |  | + |  |  |  |  |  |  |  |
| Adherence |  | + |  |  |  |  |  |  |  |
| Updated contact details |  | + |  |  |  |  |  |  |  |
| ***Central (postal or telephone)*** |  |  |  |  |  |  |  |  |  |
| Email/fax notification of allocation | x |  |  |  |  |  |  |  |  |
| Letter informing GP of participation | x |  |  |  |  |  |  |  |  |
| 1 month follow up for outpatients |  | o |  |  |  |  |  |  |  |
| 3 month prompt to patients |  |  | x |  |  |  |  |  |  |
| GP Questionnaire |  |  |  | x |  |  | x |  |  |
| Adverse events |  | o |  | x |  |  |  |  |  |
| Follow up on previous AEs |  |  |  | x |  |  | x |  |  |
| All medications |  | o |  | x |  |  | x |  |  |
| Adherence |  | o |  | x |  |  |  |  |  |
| Resource use |  |  |  | x |  |  | x |  |  |
| Patient follow up |  |  |  |  |  |  |  |  |  |
| Adverse events |  | o |  |  | x |  |  |  |  |
| Follow up on previous AEs |  |  |  |  | x |  |  | x |  |
| Adherence |  | o |  |  | x |  |  |  |  |
| modified Rankin scale |  |  |  |  | x |  |  | x |  |
| Stroke Impact Scale |  |  |  |  | x |  |  | x |  |
| Mental health inventory 5 |  |  |  |  | x |  |  | x |  |
| EQ5D-5L (HRQOL) |  |  |  |  | x |  |  | x |  |
| SF36 Vitality subscale |  |  |  |  | x |  |  | x |  |
| Resource use |  |  |  |  | x |  |  | x |  |
| Retrieve residual capsules (pill count, reconciliation and destruction) |  |  |  |  | x |  |  |  |  |

+ - only for patients enrolled as inpatients

o - only for patients enrolled as outpatients

### 4.3.1. Stroke Impact Scale [SIS]

The SIS [version 3] is 59 questions broken into 8 domains which are scored by calculating the transformed scale using the formula:

Transformed scale = [(raw score – lowest possible raw score)/possible raw score range] * 100

***I have found things which give the formula above but Im not sure if any of the questions are inverted and would good to see what the produces of scale say about calculating the score if someone has missed out a question[s].***

### 4.3.2. Mental Health Inventory 5 [MHI-5]

MHI-5 results in a score from a minimum of 5 to a maximum of 30, a higher score indicating psychological well-being and the absence of psychological distress during the past month.

To obtain the total score responses to all 5 questions are assigned a score and then summed across all questions. For questions 1 and 2 these are scored 1 point for ‘all of the time’ through to 6 points for ‘none of the time’ and for questions 3, 4 and 5 6 points are allocated for ‘all of the time’ or ‘always’ through to 1 point for ‘none of the time’ or ‘never’.

Source: <http://www.prisonmentalhealth.org/page_view.asp?c=10&did=915&fc=001007004>

### 4.3.3. EuroQol 5D-5L [EQ5D-5L]

EQ5D-5L gives a numerical value to the ‘health state’ of a person. The EQ5D-5L consists of five dimensions, each with five levels of perceived problems:

Level 1: no problem

Level 2: slight problems

Level 3: moderate problems

Level 4: severe problems

Level 5: extreme problems

Each level is assigned a numerical code [level 1 = code 1, level 2 = code 2, etc]. These dimensions are combined to give a 5 digit code with any missing dimension coded as ‘9’. Using this code we can then convert to a single index value.

EQ5D has recently been updated from 3L to 5L and at this time the producers of the measure don’t have sufficient study data to produce indexes directly so are using the ‘Crosswalk value set for EQ5D-5L’ which extrapolates from the 3L to the 5L. We expect this will be superseded with country specific values based on test data but this may take some time and may or may not be available at any point during this trial. We will use the most up to date set of index coding for this instrument at the time of analysis [http://www.euroqol.org/about-eq-5d/valuation-of-eq-5d/eq-5d-5l-value-sets.html].

Source: <http://www.euroqol.org/fileadmin/user_upload/Documenten/PDF/Folders_Flyers/UserGuide_EQ-5D-5L.pdf>

### 4.3.4. Short Form 36 [SF36] Vitality

SF36 [version 2] uses responses from 36 questions to score 8 domains, in this trial we are interested only in the vitality domain. In the full SF-36 the vitality questions are 9a, 9e, 9g, 9i. For a & e these are scored 5 points for ‘all of the time’ through to 1 point for ‘none of the time’ while g & i are scored 1 point for ‘all of the time’ through to 5 points for ‘none of the time’. A raw score is then calculated by summing across the four questions, resulting in a score from 4 to 20 with a range of 16. The raw scores are then transformed to a score ranging from 0 to 100 using the formula:

Transformed scale = [(raw score – lowest possible raw score) / possible raw score range] * 100

Source: Ware JE, Kosinski M, Dewney JE. How to Score Version 2 of the SF36 Health Survey. Lincoln, RI. Quality Metric Incorporated, 2000.

### 4.3.5. National Institute of Health Stroke Score [NIHSS]

The NIHSS is a 15 item questionnaire where responses to each question are assigned a score from 0 to 4 [some questions are scored 0 to 2 or 0 to 3 as appropriate]. The total score is calculated by summing across all questions.

### 4.3.6. Six Simple variable (SSV) model

Based on the work ‘C.Counsell, M.Dennis, M.McDowall,C.Warlow. Validation of New Prognostic Models Predicting Outcome After Acute and Subacute Stroke : Development and Validation of New Prognostic Models. *Stroke* 2002, 33:1041-1047’ we can calculate the probability of being alive and independent at 6-months using the following model:


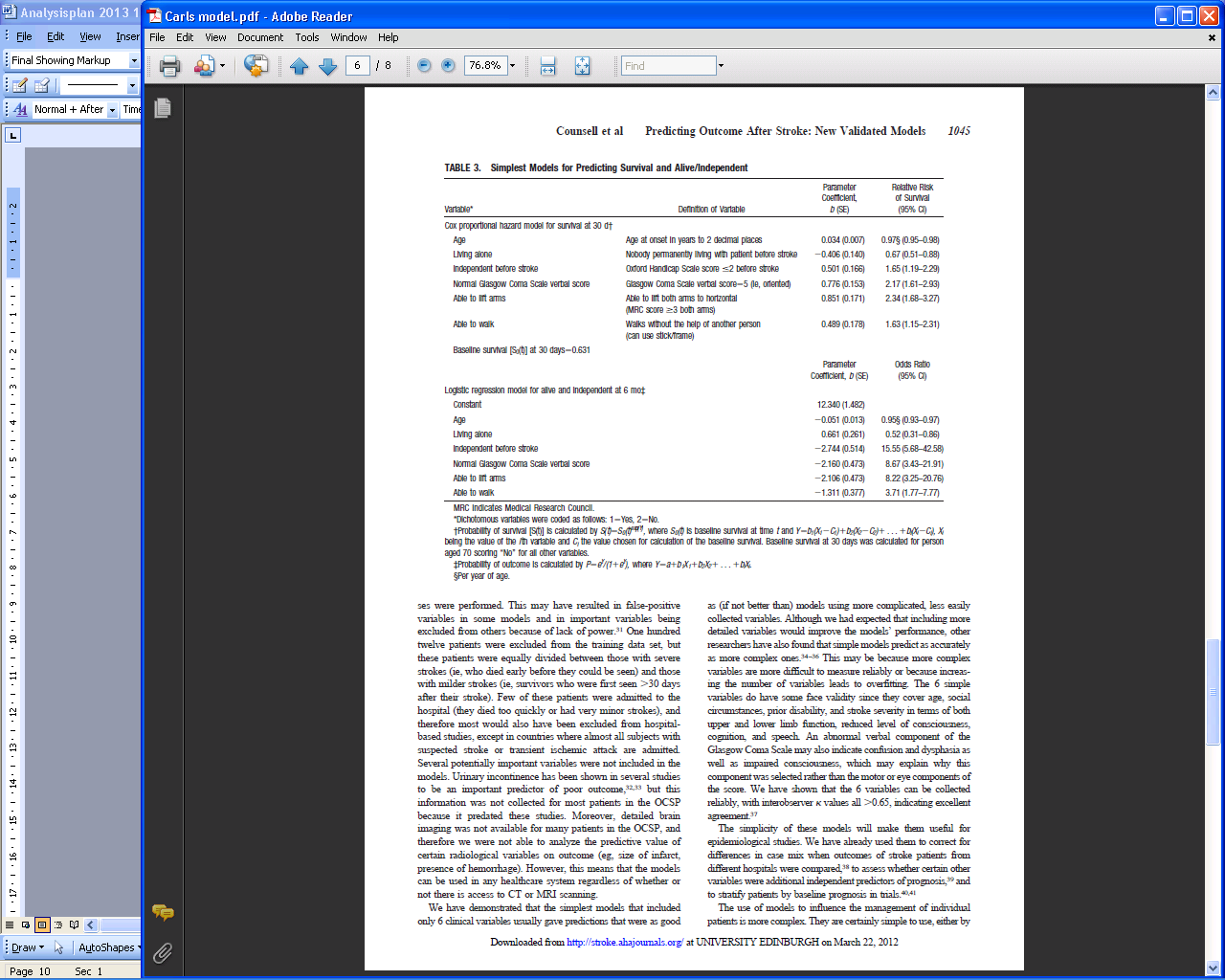


### 4.3.7. Modified Rankin Score [mRS]

The simplified version of the Modified Rankin score is based on the yes/no responses to 5 questions and is scored as follows:


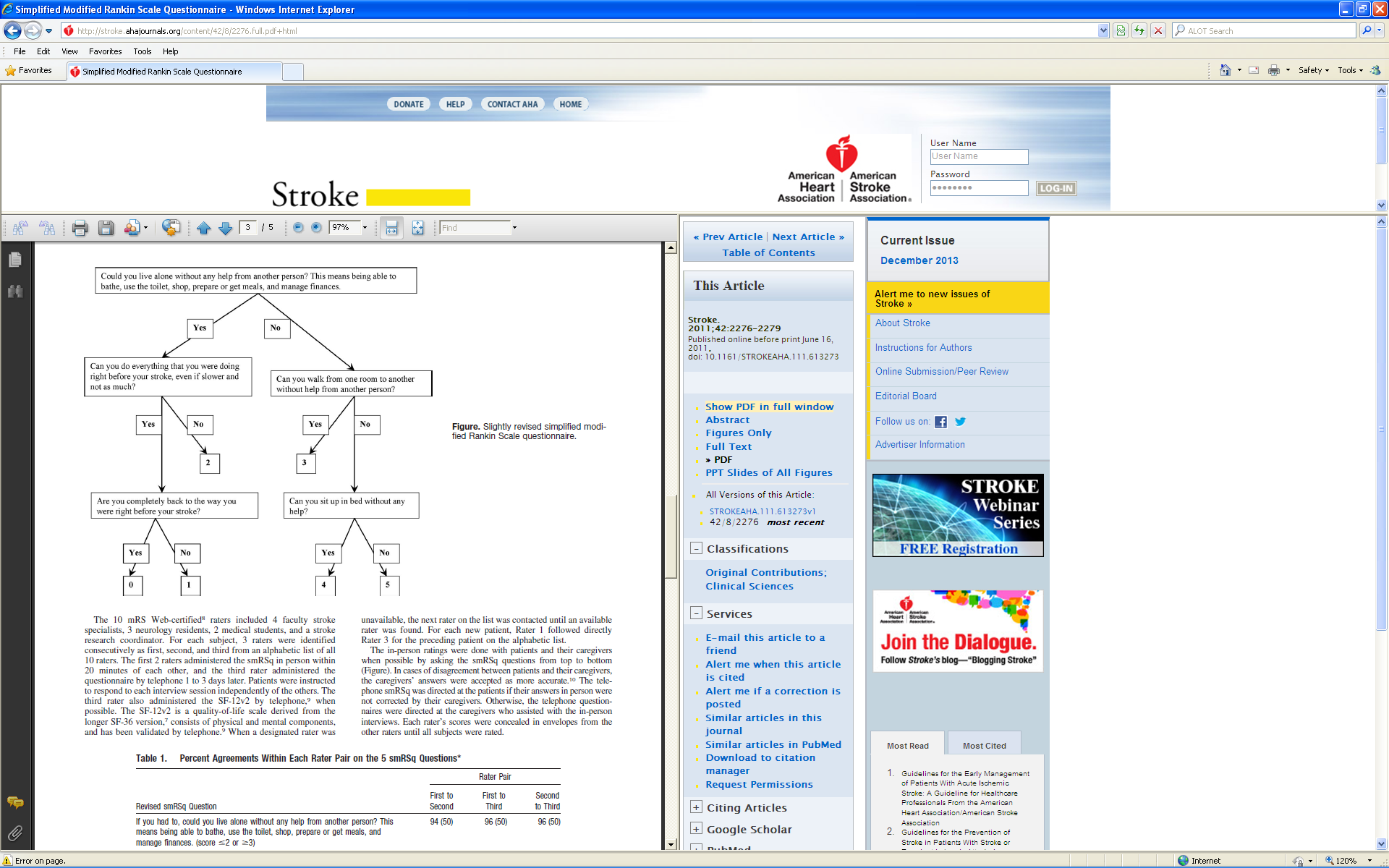


Source: Bruno A, Akinwuntan AE, Lin C, Close B, Davis K, Baute V, Aryal T, Brooks D, Hess DC, Switzer JA, Nichols FT. Simplified modified rankin scale questionnaire: reproducibility over the telephone and validation with quality of life. Stroke. 2011 Aug;42(8):2276-9

## 5. General Considerations

### 5.1. Overall

For all analyses, unless otherwise specified, we will retain participants in the treatment group to which they were originally assigned, irrespective of the treatment they actually received.

### 5.1.1. Timing of Final Analysis

The final analysis will be performed after the required number of patients has been recruited and followed up to a minimum of 12 months post recruitment. The final analysis will be performed on the data set after any ‘cleaning’ that may be required has been completed and the database locked.

### 5.1.3. Missing Data

mRS includes death so it is expected that the number with missing mRS at follow up will be minimal. Anyone with missing mRS will not be included in any analysis requiring mRS (complete case analysis).

For the quality of life measures [SF-36, EQ5D, MHI-5, SIS] we will present results for those who are alive at follow up and any discrepancy in death rates between groups will be taking into account in the interpretation. Missing data for single questions within scores will handled as detailed by each scoring method, where responses to all questions within a scale are missing that patient will not be included in that part of the analysis. If required due to amount of patients with missing scores a sensitivity analysis may be performed.

### 5.2. Interim Analysis

### 5.2.1 Interim Analysis, Data Monitoring and Stopping Rules

During the period of recruitment into the study, interim analyses of the baseline and follow up data will be supplied, in strict confidence, to the chairman of the data monitoring committee, along with any other analyses that the committee may request. In the light of these analyses, the data monitoring committee will advise the chairman of the steering committee if, in their view, the randomised comparisons have provided both (i) 'proof beyond reasonable doubt' that for all, or some, the treatment is clearly indicated or clearly contra-indicated and (ii) evidence that might reasonably be expected to materially influence future patient management.

Appropriate criteria of proof beyond reasonable doubt cannot be specified precisely, but the DMC will work on the principle that a difference of at least 3 standard errors in an interim analysis of a major outcome event (e.g. death from all causes or independent survival at six months) may be needed to justify halting, or modifying, a study before the planned completed recruitment. This criterion has the practical advantage that the exact number of interim analyses would be of little importance, and so no fixed schedule is proposed. Following a report from the DMC, the steering committee will decide whether to modify entry to the study (or seek extra data). Unless this happens however, the steering committee, the collaborators and central administrative staff will remain ignorant of the interim results.

The Chairs of the DMCs of FOCUS, AFFINTY and EFFECTS will communicate regularly to share any concerns about the accruing data and will share data if indicated. Therefore the DMC will potentially have access to all available information when making its recommendations. This aims to maximise patient safety.

### 5.2.2 Interim Analysis and Sample Size Adjustment

The trial steering committee (TSC) will review the target sample size and adjust this based on:

- Advice from the DMC
- Accruing data on
  - the enrolment into specific pre-specified subgroups
  - completeness of follow up
  - distribution of mRS categories in the population of enrolled subjects (i.e. both treatment groups combined), overall and in specific patient categories (e.g. those with motor deficits, aphasia, etc)

For example, if the distribution of mRS is different to that anticipated, then the sample size might need to be increased. This approach has the advantage that such sample size adjustments can be made without reference to the accumulating unblinded data, and avoids the need for conditional power calculations which can be unreliable.

### 5.2.3. Practical Measures to Minimise Bias

In producing the DMC report the trial statistician will be unblinded to treatment allocation although the primary outcomes will be verified by a second statistician who will be aware of which group participants are in but not what treatment each group received. In addition to that Dr Steff Lewis will be involved as a blinded statistician and any statistical issues that require to be addressed through the course of this trial will be considered by her in a blinded manner.

### 5.2.4. Other evidence

Any relevant updated systematic reviews will be circulated to the DMC, and the results will be added into a systematic review at the end of the trial. There is one relevant Cochrane review: Mead GE, Hankey GJ, Kutlubaev MA, Lee R, Bailey M, Hackett ML. Selective serotonin reuptake inhibitors (SSRIs) for stroke. Cochrane Database of Systematic Reviews 2011, Issue 11. Art. No.: CD009286. DOI: 10.1002/14651858.CD009286

## 6. Summary of DMC Report Data

All categorical data [including binary data] will be presented as numbers and percentages broken down by randomised treatment. Where data are continuous descriptive statistics will be presented [N, mean, sd, min, max, median, Q1, Q3] broken down by randomised treatment.

### 6.1. Recruitment

Standard accrual plot, and plot of number of patients recruited each quarter. Table showing number of centres recruiting patients.

### 6.2. Trial randomisation flow chart

For each form, % received, % not received + reasons why forms missing.

### 6.3. Success of minimisation, and baseline data

**Minimisation**: Check minimisation is successfully balancing on minimisation factors.

**Other baseline data [collected on randomisation form]:** Check treatment balanced by sex, ethnicity, whether patient is an inpatient, depression, diabetes, previous CHD, previous ischaemic stroke/TIA, previous intracranial bleeding, history of GI bleeding, hyponatraemia, bone fractures, current mood [both yes, 1 yes, none yes], recent intracerebral bleeding, type of stroke/OCSP classification and total NIHSS.

**Other data collected post treatment [collected on discharge form]:** Check the following are equally balanced by treatment group: final diagnosis of randomising event, post randomisation non-trial medication, discharge destination and in-hospital compliance variables for inpatients [received any trial medication, taking till death/discharge, reasons for not taken till death or discharge and doses missed].

### 6.4. Protocol violations, compliance and blinding

**Inclusion/Exclusion violations:** quantify the number and % of participants randomised >15 days after stroke onset, diagnosis of subarachnoid haemorrhage

**Compliance**: Each participant will be issued with 6 months supply of trial medication, at 6 months they will be asked if they have taken all capsules, asked on average how often they took capsules and asked the reasons for stopping as well as date of stoppage.

Our main measure of compliance will be the patient reported completion of medication. However at the end of each participants involvement any unused medication will be returned and counted. A check on the patient reported compliance will be determined based on the number of pills not returned and the duration each participant has spent in the trial.

**Blinding:** quantify the number of patients who required unbinding of study medication during the trial by treatment arm and where available present the reasons for unblinding.

### 6.5. Adverse events

Adverse events will be analysed by treatment as received, not by treatment as allocated. Any adverse event occurring during initial in-patient stay should be recorded on the discharge form: further stroke, acute coronary event, upper GI bleed, fall with injury, new fractures, definite epileptic seizure, symptomatic hypoglycaemia, hyperglycaemia, hyponatraemia, new depression [diagnosis/treatment], new antidepressants given, suicide/self harm.

Post discharge the same list of events will be captured as part of the patient update. Any serious adverse events will be captured and information presented by treatment received.

In hospital and post discharge AEs will be quantified and presented as number and % of patients broken down by treatment received.

### 6.6. Outcomes

**Primary outcome:** Number and percent of patients in each treatment group will be presented by ordinal mRS. Odds ratio, 95% CI for odds ratio will be presented for both adjusted and unadjusted analysis, as described for the final analysis. Adjustment will only be performed if sufficient data have accrued.

**Secondary outcomes:** For binary outcomes odds ratio, 95% CI for odds ratio will be presented for both adjusted and unadjusted analysis, as described for the final analysis. Adjustment will only be performed if sufficient data have accrued.. For continuous outcomes descriptive statistics will be presented [N, mean, sd, min, max, median, Q1, Q3] broken down by randomised treatment.

## 7. Final analysis

### 7.1. Primary outcome

Comparison of Modified Rankin score [mRS] at six-months post randomisation by treatment group where mRS treated in its full ordinal format [scores of 0,1,2,3,4,5,6]. This will be examined using an ordinal logistic regression and will be performed in both an unadjusted manner and also adjusted for factors in the baseline minimisation (as recommended by the Draft CPMP guidelines, December 2001). We will present common odds ratios using the proportional odds method and for the unadjusted analysis we will also present the individual dichotomies.

### 7.2. Secondary outcomes - binary

Where the outcome of interest is binary, comparison by treatment group will be examined using a binary logistic regression and will be adjusted for factors used in the minimisation algorithm, unadjusted odds ratios will also be presented. In addition the results will be presented as relative risks with 95% CI, calculated from the odds ratio [using methods described by Deeks JJ, Higgins JPT, Altman DG (editors). Chapter 9: Analysing data and undertaking meta-analyses. In: Higgins JPT, Green S (editors). Cochrane Handbook for Systematic Reviews of Interventions. Version 5.0.1 [updated September 2008]. The Cochrane Collaboration, 2008. Available from [www.cochrane-handbook.org](http://www.cochrane-handbook.org/).] and absolute risk differences with 95% CI.

1. Modified Rankin score at six-months [mRS 0-2 v mRS 3-6].
2. Death from any cause by six-months.
3. Death from any cause by twelve-months.
4. New diagnosis of depression since randomisation by six-months.
5. New diagnosis of depression since randomisation by twelve-months.

### 7.3. Secondary outcomes - ordinal

Modified Rankin score at twelve-months will be examined in its full format [scores of 0,1,2,3,4,5,6] using the methods described for the primary outcome.

### 7.4. Secondary outcomes - continuous

Where the outcome of interest is continuous, these will be compared between treatment groups using linear regression adjusting for factors in the baseline minimisation, or an alternative approach as necessary. This analysis will be conducted for the following outcomes at both six and twelve months.

1. Quality of life as measured by the EuroQol [EQ-5D]
2. Depression and anxiety as measured by the mental health inventory 5 [MHI-5 – question 21,22 of 6-month follow up; question 17,18 of 12-month follow up] and SIS [question 14 on 6-month follow up; question 10 on 12-month follow up].
3. Fatigue as measured by the vitality sub-scale of the Short Form (36) Health Survey [SF-36 question 20 on 6-month follow up; question 16 on 12-month follow up].
4. Limb strength as measured by SIS [question 12 on 6-month follow up; question 8 on 12-month follow up].
5. Hand function as measured by SIS [question 18 on 6-month follow up; question 14 on 12-month follow up].
6. Cognitive function as measured by SIS [question 13 on 6-month follow up; question 9 on 12-month follow up].
7. Communication as measured by SIS [question 15 on 6-month follow up; question 11 on 12-month follow up].
8. Extended ADL as measured by SIS [question 16 on 6-month follow up; question 12 on 12-month follow up].
9. Mobility as measured by SIS [question 17 on 6-month follow up; question 13 on 12-month follow up].
10. Social activity as measured by SIS [question 19 on 6-month follow up; question 15 on 12-month follow up].
11. Overall patient outcome as measured by the SIS total score
12. Overall recovery as measured by SIS thermometer [question 28 on 6-month follow up; question 24 on 12-month follow up].

### 7.5. Secondary outcomes – survival

Survival over the study period will be examined using Cox proportional hazard model adjusted for factors in the minimisation algorithm with survival curve going beyond 12m if there is enough data available according to Pocock SJ, Clayton TC, Altman DG. Survival plots of time-to-event outcomes in clinical trials: good practice and pitfalls. *Lancet* 2002;**359**:1686-9.

### 7.6. Pre-specified sub group analysis

Comparison of mRS at 6-months based on Ordinal analysis will be split by the following sub-groups: age [<70, ≥70], baseline probability of being alive and independent at 6-months [<0.15, 0.16-1 based on Carls model], type of stroke [ischaemic, haemorrhagic], patient able to provide own consent [yes, no] adjusting for baseline motor impairment [yes/no], baseline aphasia [yes/no] and baseline depression [yes/no].

Motor impairment: In patients with motor impairment resulting from the initial stroke [where motor impairment is defined as above] is there a difference in the motor function at 6 months [as measured by SIS, question 12, 17, 18 on follow up)]. Is this maintained at 12 months? Linear regression analysis will be performed on the baseline to 6-month change and separately for the 6-month to 12-month change both unadjusted and also adjusted for factors in the minimisation.*.*

Communication impairment: In patients with communication impairment resulting for the initial stroke [where communication impairment is defined as above on NIHSS] is there a difference in the communication function at 6 months [as measured by SIS, question 15]. Is this maintained at 12 months? This analysis will be performed unadjusted and also adjusted for factors in the minimisation.

Linear regression analysis will be performed on the baseline to 6-month change and separately for the 6-month to 12-month change both unadjusted and also adjusted for factors in the minimisation.

Mood: Is there a relationship between outcome [as defined by mRS at 6-months] and mood [measured using MHI5] and is this relationship affected by Fluoxetine?

### 7.7. Possible extra analyses

AFFINITY, EFFECTS and a Polish study are all running simultaneously to FOCUS, at the conclusion of these trials an individual patient meta-analysis may be carried out.

## 8. Revision History

| **Document version & date** | **Review comment** | Date of review | **Reviewer’s Signature** |
| --- | --- | --- | --- |
|  |  |  |  |
